# The Gentleman Artist-Surgeon in Late Victorian Group Portraiture

**DOI:** 10.1080/14714787.2013.780828

**Published:** 2013-04-22

**Authors:** Keren Rosa Hammerschlag

**Keywords:** group portraiture, surgery, Victorian, gentleman, Henry Jamyn Brooks (1865–1925), Solomon Joseph Solomon (1860–1927), Henry Thompson (1820–1904)

## Abstract

In this article I consider the ways in which group portraits of surgeons, a genre associated with inscriptions of corporate membership and institutional authority, reflected the complex and at times contradictory status of surgeons during the late Victorian period. Group portraits from this period offer a diverse range of representations of surgeons – from middle-class professional to hygiene reformer, scientist to cultured gentleman – all of which worked against the popular conception of the surgeon as manual labourer and bloody carpenter. In particular, the emergence during the period of the gentleman artist-surgeon, exemplified by the celebrity surgeon and amateur artist Henry Thompson (1820–1904), signalled a new incarnation of the surgeon and offered an alternative to both the stereotypes of the surgeon as manual labourer and the surgeon or middle-class professional. But there were complexities and contradictions that beset the identity of the gentleman artist-surgeon, and these will be considered with reference to Thompson’s own novel, *Charley Kingston’s Aunt* (1885).

## Introduction

In 1885 the celebrity surgeon and amateur artist Henry Thompson (writing under the pseudonym Pen Oliver) published a novel entitled *Charley Kingston’s Aunt*. In it, a medical student, Charley, discovers that the corpse that he has been dissecting is that of his beloved ‘old nanny’. The novel betrays ambivalence on the part of Thompson towards the practice of dissection, the narrator acknowledging that it still generates in most people ‘profound repugnance’ and ‘a half-suppressed wish that no such practice as dissection existed’.^1^ In what constitutes an awkward solution to the problem of dissection and Charley’s lack of financial security, in the end it is the dissected corpse of Charley Kingston’s aunt that provides the inheritance that ensures the surgeon’s elevated social status.

In this article I analyse the development during the late nineteenth century of the genre of group portraiture – a genre which has received near to no attention in art historical scholarship since Alois Riegl’s *The Group Portraiture of Holland* (1902) – in order to trace the shifting representation of surgeons in relation to the rise of the professional classes.^2^ By the end of the century, surgeons had managed to join the ranks of the Victorian middle classes. Yet they continued to be dogged by the popular perception of surgery as rough, bloody work. I therefore consider the ways in which group portraits of surgeons inscribed corporate membership and institutional authority, while reflecting the complex and at times contradictory status of surgeons during the late Victorian period. I show that portrait painters both drew on existing conventions for depicting surgeons as scientists and developed new modes by which to establish them as middle-class professionals and cultured gentlemen. Several scholars have offered accounts of the important place of the visual in Victorian science.^3^ This article will address the significance of representations of science, especially anatomy, in Victorian portraiture, drawing on Ludmilla Jordanova’s contribution to the subject in *Defining Features: Scientific and Medical Portraits 1660–2000*.^4^

I take as my focus three diverse examples, namely Henry Jamyn Brooks’ *Council of the College, 1884–85* (1887) and *The Viva* (1894), and Solomon Joseph Solomon’s *An Octave for Mr Ernest Hart at Sir Henry Thompson’s House* (*c*. 1897). None of these paintings have received any focused scholarly attention, and yet they offer three rich case studies of different artistic constructions of surgeons from the period. I argue that these group portraits actively constructed surgeons as professionals, scientists and art connoisseurs respectively. At the same time, although all these roles worked against the stereotype of the surgeon as bloody barber and hangman, and countered associations of surgeons with the Burke and Hare scandal of the 1830s,^5^ there were tensions among them, especially in terms of the divergent models of masculinity they represented. Recent studies of Victorian masculinity have shed light on the procedures of male self-styling, especially in the case of dandies and aesthetes.^6^ I seek to extend existing masculinity studies to address the particularly problematic case of Victorian surgeons. In particular, I identify the emergence during the period of the gentleman artist-surgeon, most thoroughly exemplified by Henry Thompson (1820–1904), which signalled a new incarnation of the surgeon and offered an alternative to the figure of the middle-class professional. But while surgery was made less barbaric with the advent of anaesthesia, the work of surgeons remained far from gentlemanly. As Mrs Carey recalls in Somerset Maugham’s *Of Human Bondage* (1915), she ‘did not forget that in her young days no one ever considered the doctor a gentleman’.^7^

## Distinguishing surgeons: *Council of the College, 1884–85*

Riegl argues in *The Group Portraiture of Holland* that paintings of surgeons congregating around skeletons or dissecting tables were some of the earliest forms of group portraiture.^8^ Henry Jamyn Brooks’ *Council of the College, 1884–85* (1887, Figure 1) can therefore be located within an artistic tradition dating back to Rembrandt’s *Anatomy Lesson of Dr Tulp* (1632). As in other Victorian portraits and group portraits of surgeons, however, in Brooks’ *Council of the College* there is a conspicuous absence of any indicators of actual surgery. No painting comparable to Thomas Eakins’ *The Gross Clinic* (1875) came out of the English context, and even in its American context *The Gross Clinic* was vehemently criticized for its graphic treatment of the subject of surgery.^9^ In France, numerous important paintings of surgeons pictured them in anatomy or operating theatres. For example, Henri Gervex’s *Avant l’op*é*ration* (1897) depicts Jules Emile Péan lecturing at the Saint-Louis hospital and demonstrating his homeostatic clamps. Nevertheless, as Mary Hunter has argued, while in Gervex’s painting Péan is presented as a surgeon rather than an anatomist, he is not depicted in the act of surgery.^10^ She notes that ‘Gervex depicted the moment before the operation, the moment of cleanliness, hygiene, and control, the moment before the surgeon’s clean hands and white shirt would be stained with the woman’s blood’.^11^

*Council of the College, 1884–85* was the first group portrait to be produced of the councillors of the Royal College of Surgeons in a format that would be repeated at various intervals until the present day. It is my contention that this group portrait and those like it that followed reflect both the increased professionalism of surgeons during this period and surgeons’ need to inscribe, construct and affirm their professionalism. After all, any account of the class ascendancy of doctors during the nineteenth century needs to be underscored by an acknowledgement of the precariousness of their middle-class standing. This point is made by Jongwoo Jeremy Kim when he states that ‘[p]rofessional men such as doctors, barristers, vicars, and military officers entered the middle classes by dint of their knowledge and skills, but, among these men, those who did not possess land-based hereditary wealth were often subjected to the ever-present anxiety of losing their social stations’.^12^

Brooks painted numerous group portraits during the nineteenth century, including *Private View of the Old Masters Exhibition, Royal Academy 1888* (1889; National Portrait Gallery), a painting of the most powerful members of the late Victorian art world.^13^ A consideration of *Private View* in relation to *Council of the College* invites reflection on the similarly precarious class status of artists and surgeons during this period, both having succeeded in joining the professional classes but whose gentlemanly standing nonetheless remained precarious owing to the continued association of their occupations with manual labour.^14^

Brooks described the making of *Private View* in an unpublished manuscript entitled ‘The Dilettante’ (1914), which provides invaluable insights into how he produced his complex group portraits.^15^ Significantly, his working method involved selecting subjects and developing a composition independent of the event depicted. He used photography as well as life sittings for the execution of likenesses. He also, on occasion, resorted to using models when organizing a composition, writing that ‘[a]s might be expected, when each person was drawn upon the canvas they did not all fit in, so in many cases I had to change the position and redraw the figure from a model or from the sitter as circumstances permitted’. Brooks’ acknowledgment that each person did not necessarily ‘fit in’ reflects the strange procedure of arranging individual portraits into a cohesive scene involved in the production of group portraits.

**Figure 2:**
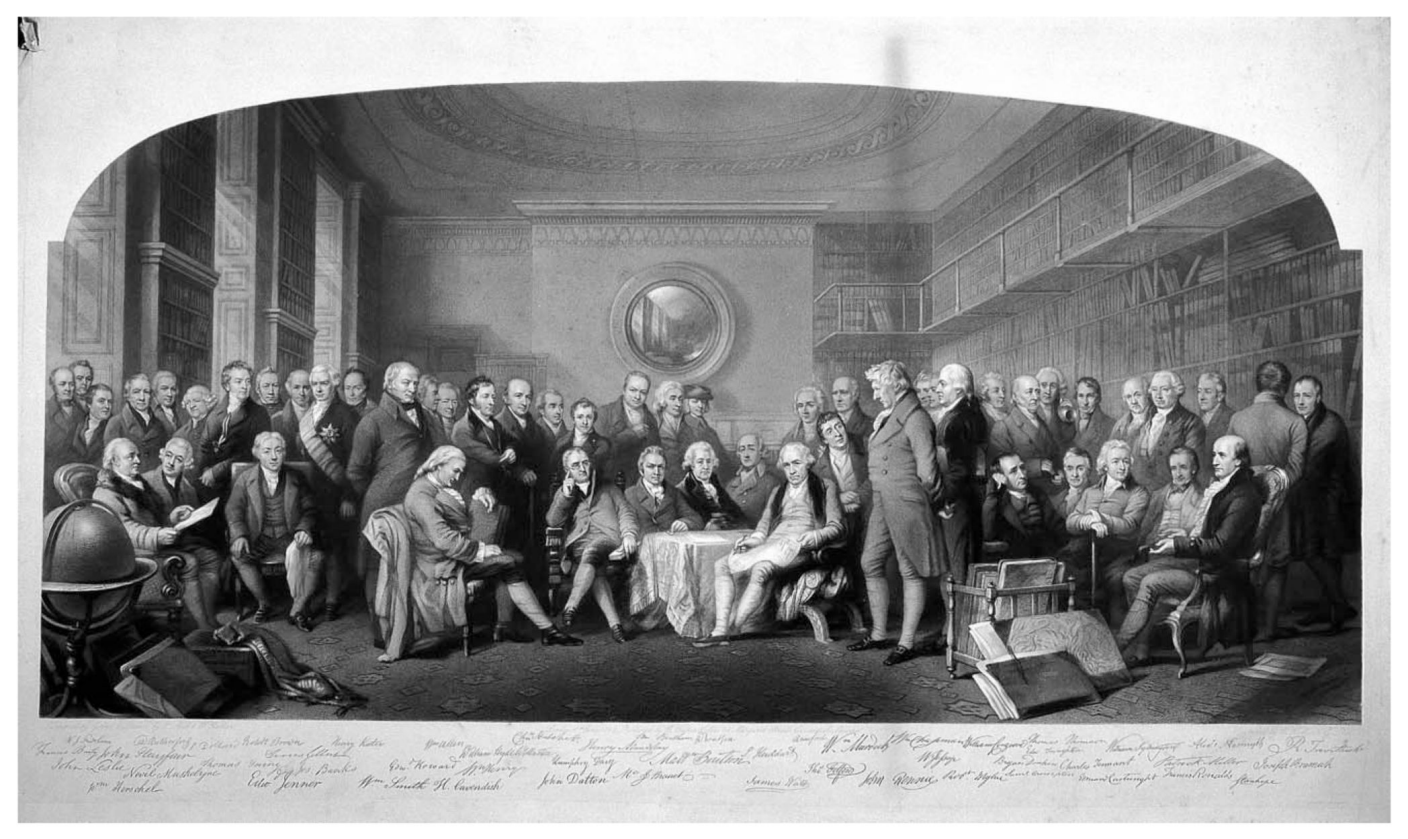
John Gilbert after William Walker, *The Distinguished Men of Science of Great Britain, Living in 1807/8*, *c*. 1860, platemark 65.6 x 111.6 cm. Wellcome Library, London.

The composition *Council of the College, 1884–85* resembles William Walker’s widely reproduced engraving of 1862, *The Distinguished Men of Science of Great Britain, Living in A.D 1807–8* (Figure 2).^16^ In particular, the disorganized pile of books in the foreground of Brooks’ painting recalls the placement of the globe, books and other items in Walker’s engraving. Together Walker and Brooks offered a history of British scientific achievement stretching across the nineteenth century from the vantage point of the century’s second half. But Walker included only one surgeon in his invented community of distinguished men of science: Edward Jenner, who was famous for his pioneering work on the smallpox vaccination. In other words, Walker largely excluded surgeons, including high-profile examples (such as Astley Paston Cooper) who were alive in 1807–8.^17^

By appropriating Walker’s composition, Brooks’ *Council of the College* constructed British surgeons as ‘distinguished men of science’. The inclusion of the pile of books in the painting’s foreground denotes the surgeons’ intellect and learning.^18^ While we cannot identify exactly which books they are, their hefty size and resemblance to those in Walker’s engraving might lead one to conclude that they are tomes of classical scholarship, attributing to the surgeons the classical education of physicians and gentlemen, or books on the body by Vesalius and Cheselden.^19^

**Figure 3:**
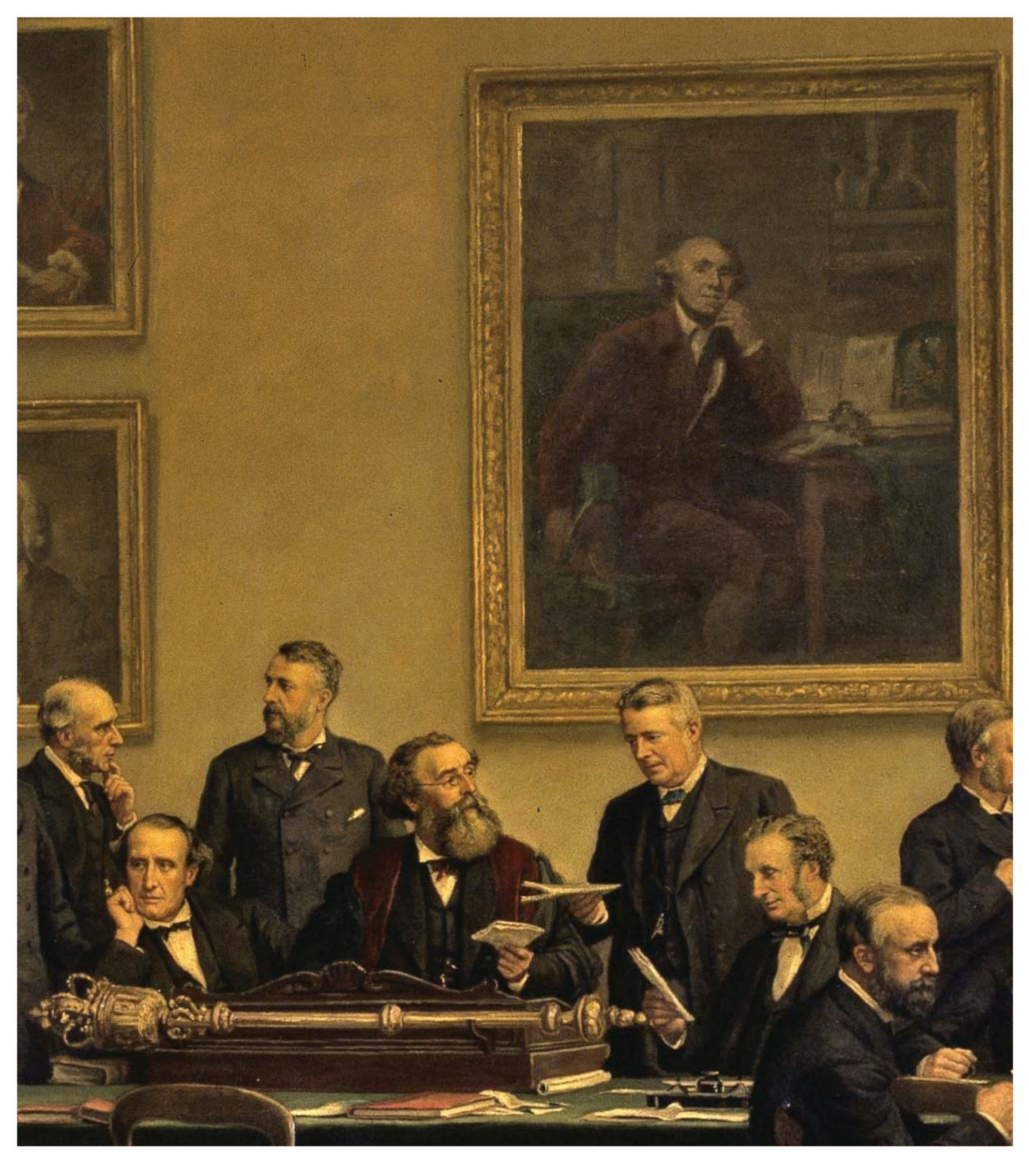
Detail from Henry Jamyn Brooks, *Council of the College, 1884–85*, 1887, oil on canvas. Courtesy of the Royal College of Surgeons of England.

In *Council of the College*, Brooks drew on conventions for depicting surgeons as scientists which dated back to the eighteenth century and which were associated, above all, with Joshua Reynolds’ portrait of John Hunter (1785). As Ludmilla Jordanova has shown, such conventions were deployed in order to associate doctors and surgeons with learning and scholarship rather than manual labour or the barbarism of surgery and dissection.^20^ In Brooks’ painting the surgeons stand and sit in front of hung portraits which place them within a lineage of esteemed men of their profession – Reynolds’ portrait of Hunter appearing most prominently in the centre of the composition. Aris Sarafianos writes that ‘[f]rom 1817 onwards, when it passed from the Hunter family to the Royal College of Surgeons, Reynolds’s portrait featured at the centre of the organisation’s public image’.^21^ In the same year as Brooks painted *Council of the College*, William Savory, Vice President of the Council, delivered an oration in praise of Hunter, crediting him with raising surgery from the barber’s craft to a scientific art. In Brooks’ painting, Savory rests his head on his hand in a gesture that connotes thoughtfulness, but which also repeats in reverse the pose of Hunter in Reynolds’ portrait (detail, Figure 3). A direct visual connection is therefore established between surgeons past and present.

While drawing on traditions in portraiture, especially those associated with eighteenth-century portraits of scientist-surgeons, *Council of the College* signalled the emergence of a new type of corporate group portraiture, which made visible the professional status and gentlemanly standing of surgeons. Thomas Spencer Wells, President of the Council from 1882 to 1883, presented Brooks’ *Council of the College* to the Royal College of Surgeons in 1887 on behalf of the subscribers who financed its production.^22^ One can assume that Wells directed Brooks as to whom to include and where to place them. The painting features representations of some of the Victorian period’s most eminent surgeons: Joseph Lister, John Marshall, James Paget and, of course, Wells. Their uniformity of dress, comportment, gender, class and race attest to the fact that these surgeons were members of a privileged professional corporation. In particular, their black suits enforce, in the words of John Harvey, ‘a certain sombre gravity of power’.^23^

**Figure 4:**
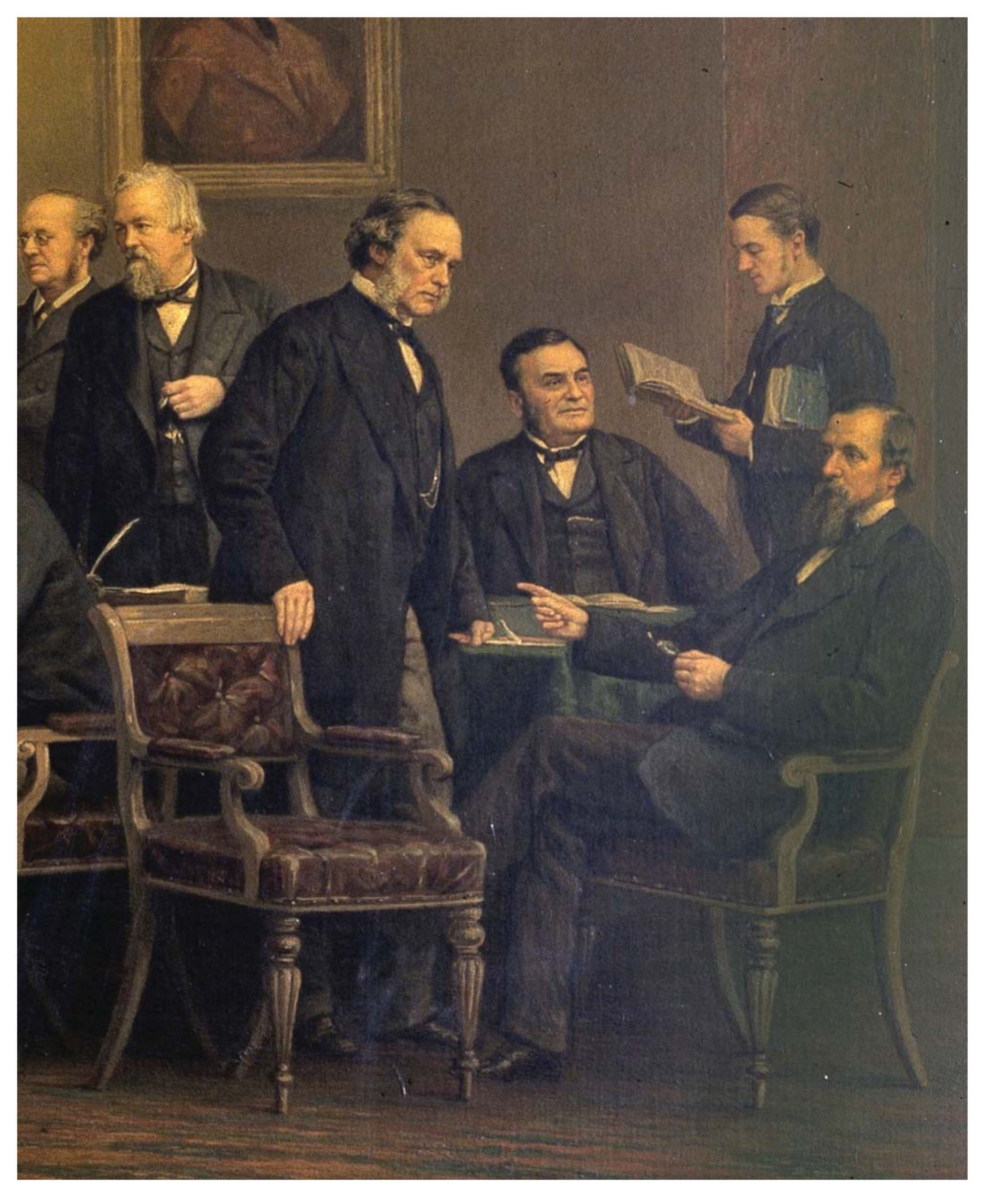
Detail from Henry Jamyn Brooks, *Council of the College, 1884–85*, 1887, oil on canvas. Courtesy of the Royal College of Surgeons of England.

At the same time, individual surgeons are distinguished through facial features, hand gestures and their placement in the composition. The President of the Council, John Cooper Forester, sits in the middle of the group – the modern-day Christ presiding over the *Last Supper –* unmovable in his triangulated body shape and differentiated by his red gown that is visually echoed in the red of the portraits behind him. Wells sits two places to the President’s right. He refrains from engaging in discussion with other members of the group and, in so doing, is separated out from the general assembly. He also appears against a blank wall rather than in front of other men, which further distinguishes him from the crowd. The senior surgeon, James Paget, stands to the right of Wells and is the only figure to look out directly towards the viewer in much the same way as he does in the 1872 portrait of him by John Everett Millais. Finally, Lister (detail, Figure 4) stands apart from the surrounding clusters while Jonathan Hutchinson points at him, directing the viewer’s eye to the esteemed inventor of antiseptics. Even in the context of a group portrait, individual innovation is constructed as requiring a degree of separation from the group.

The placement of figures also reveals political alliances and divisions among the surgeons. In 1745 the Company of Surgeons broke away from the Barber-Surgeons’ Company; in 1800 it obtained a royal charter and became a Royal College; and in 1845 its jurisdiction was extended to cover the whole of England.^24^ In the 1880s the movement for obtaining greater privileges and power for Fellows and Members began to gain momentum.^25^ Brooks’ painting might document specific council meetings that took place during the years 1884 and 1885 at which votes were taken on several important resolutions relating to the corporate organization and running of the College. For example, on May 8, 1884, the councillors passed a resolution allowing Fellows and Members to hold annual meetings, but rejected the recommendation that Fellows elect the President of the College.^26^

Lister was one of only five councillors who supported having Fellows vote for the President of the College at the May 8 meeting.^27^ This is unsurprising considering that Lister’s antiseptic techniques were not initially embraced by the older generation of British surgeons who made up the majority of the council, especially during the years 1884–5.^28^ Savory publicly opposed Lister in an address delivered at the British Medical Association in 1879. In Brooks’ painting, Lister’s status as sidelined and contrary is suggested through his turning his back on the majority of councillors as though he is walking away. He is couched between John Marshall (standing) – one of the early advocates of Listerian antisepsis – and John Wood (seated), who was lecturer of clinical surgery jointly with Lister at King’s College Hospital.^29^ Marshall and Wood, it seems, were content to be pictured in Lister’s proximity.

## Examining surgeons: *The Viva*

Whereas Brooks’ painting of the councillors depicts surgeons who had already been admitted into the inner sanctum of the Royal College of Surgeons, his later group portrait, *The Viva* (1894, Figure 5),^30^ depicts the procedures involved in gaining admission to the profession.^31^ It features representations of recognizable surgeons – John Wood and Jonathan Hutchinson at the front table; Edward Lund and William MacCormac to the left; and John Marshall and Frederick Le Gros Clark on the right – all but Clark having appeared in *Council of the College*. They are shown administering an examination and thereby granting (or denying) the next generation of surgeons access into the profession.^32^ The painting illustrates the increased importance of medical corporations, such as the Royal College of Surgeons, in examining and certifying candidates’ fitness to practise medicine.^33^ It attests to the rigorous training and stringent examinations required of surgeons and emphasizes the difficulty of gaining admission to surgery’s professional institutions.^34^

Thompson described an oral examination for admission to the Royal College of Surgeons in *Charley Kingston’s Aunt.* He noted that the examination ‘consisted solely of questions personally addressed to the candidate during the short term of one hour by several different examiners in succession’, and that ‘each examiner was more or less a surgical celebrity’.^35^ He wrote: ‘[A]t each table sat two examiners, and one vacant chair … Four students at a time were ushered in with ceremony. One was conducted to each table, and each remained there receiving and answering questions.’^36^ That Brooks depicted an oral examination with single candidates being examined by two surgical celebrities corroborates Thompson’s description, suggesting that Brooks’ painting is an accurate record of an actual event. By the time of the painting’s execution, however, three of the surgical celebrities – Marshall, Wood and Clark – were dead. It is possible that Brooks used studies and photographs from *Council of the College* for the production of *The Viva*. The painting seems to have been commissioned by Hutchinson as a retrospective group portrait, intended to memorialize the experience of examining with his late friend, Wood.

As in the previous group portrait by Brooks, objects are included in order to indicate the surgeons’ learning and knowledge. A large folder, presumably with demonstrative anatomical diagrams, is positioned next to the examiners in the foreground. Books lie on the floor behind the examiners to the right of the picture. These items are carefully positioned near to the senior surgeons as opposed to the younger candidates, thereby attributing the possession of knowledge to them, rather than to the aspiring surgeons who have yet to prove their credentials. It is also possible that they are books that Marshall had written, such as *A description of the human body: its structure and functions designed for the use of teachers in schools and young men destined for the medical profession, and for popular instruction generally* (1860).^37^

The painting entered the Royal College of Surgeons’ collection in 1948, having previously been housed in the Haslemere Museum in Surrey, which Hutchinson established in 1894 as an educational museum of specimens scientifically arranged for methodical instruction and study, and before that in Hutchinson’s educational museum in Shelby.^38^ I believe that *The Viva* had an educational purpose: to guide viewers at an educational museum as to the right and wrong way to examine the specimens on display.^39^ From as early as 1885, Hutchinson expressed the view that ‘educational museums and the improvement of educational examinations’ were ‘two of the most important features in the development of objective teaching’.^40^ He even went so far as to institute museum examinations for children, which required the close study of items in the collection and demanded the ‘identification on the spot of certain miscellaneous objects and portraits taken from the museum’.^41^

In both museum education and medical education, Hutchinson advocated the intimate handling of specimens in order to accumulate knowledge. In a speech given at the opening of the Museum of Antiquities and Natural Curiosities in Newbury in 1904, he stated that educational museums were ‘designed to accumulate evidence of a kind which is likely to make pleasurable, clear and deep impressions. The objects contained in them are not only to be seen, but when suitable, to be handled.’^42^ He advised that curators of educational museums should ‘avoid all expensive objects’ and ‘rarities’, displaying instead ‘objects which can be handled without damage and replaced without much cost’.^43^ Furthermore, according to Hutchinson, the handling of objects was required in the context of museum and medical examinations. In 1895 he said of objective examinations that ‘objective’ meant ‘the inspection, identification, and description of things’, and that ‘[i]n Geology, for instance, a man could not offhand identify the spine of an echinus or distinguish the shells of a brachiopod from the true bivalve unless he had seen and handled such objects before’.^44^

In Brooks’ picture of a medical examination, the prospective surgeon at the table to the painting’s right uses his sense of touch to identify his specimen, actively handling it in much the same way as visitors to the Haslemere Museum were invited to handle the objects on display. Rather than just looking at the specimen, as does the prospective surgeon in the background to the left, the student holds and feels it, engaging in a seemingly more successful mode of identification. We are also invited to compare the ways in which the student in the foreground and the student to the back left examine their specimens. Both deploy their sense of vision, but the figure in the foreground is depicted as actively investigating his specimen.

It is possible that the surgical student in the foreground was modelled on Victor Horsley (1857–1916), son of the artist John Callcott Horsley, as is suggested by his distinguishing handlebar moustache and the flamboyant manner in which he examines his specimen.^45^ Furthermore, he is depicted examining a brain in a jar, an appropriate choice for one of the pioneers of modern neurology. By the time of the painting’s execution, Horsley was well established as a leader in the field of cerebral surgery, having performed some of the first successful operations of their kind.^46^ The presentation of Horsley as a student, therefore, indicates the painting’s status as nostalgic recreation.

Artworks appear prominently in this scene of anatomical investigation, indicating the presence of art within surgical training. They also allude to the importance of anatomical knowledge for artistic renderings of the human form. On the mantel there are classical statuettes – the *Apollo Belvedere* and *The Dancing Faun* – two classical male nudes, which were used in the study of anatomy by both surgeons and artists during the nineteenth century.^47^ The inclusion of these statuettes reminds the viewer of medicine’s and art’s shared investment in the visual, and the ways in which both medical diagnostics and art making, especially sculpture, engage the tactile.^48^

As in *Council of the College*, portraits of surgeons past appear in *The Viva*. They allude to the lineage of surgeons to which the students are attempting to gain access and who representationally oversee their examination. On the left is the portrait of Anthony Carlisle by Martin Archer Shee (1824); on the right is Astley Paston Cooper by Thomas Lawrence (1828); the central portrait, however, is so severely truncated that it is difficult to attribute firmly.^49^ Like Hunter, these surgeons were celebrated as learned men of science, specifically of anatomy.^50^ In fact, it is those signifiers of scientific learning – the skull, paper and quill, and books – that remain visible even after the paintings have been cropped by Brooks’ composition.

But the inclusion of portraits in a painting intended for Hutchinson’s educational museum holds further significance. Hutchinson established a portrait gallery in the Haslemere Museum because he believed that ‘[t]he study of the features of the human face is one of universal interest, and in many ways likely to prove elevating and profitable’.^51^ Students undertaking his museum examinations were required to identify personages in portraits as well as objects from the natural sciences. Like the prospective surgeons in Brooks’ painting who are required to identify body parts on the basis of the close examination of anatomical specimens, and the students at the Haslemere Museum who were required to identify portraits as part of their museum examinations, we viewers are challenged to identify the surgeons in the portraits on the basis of the body parts and props provided. Because the sitters are compositionally decapitated by the top edge of the canvas, however, these are portraits without faces. In turn, our ability to identify them accurately is problematized.

In the identification of anatomical and pathological specimens, as with the identification of sitters in portraits, diagnostic looking is required, but it is also liable to error and misrecognition, especially when it is only a partial body that is in view. As pictured in *The Viva*, in order to become surgeons, the students have to examine the anatomical specimens and identify them correctly. At the same time, the surgeons in the scene (and the modern viewer outside it) examine the students in order to assess who might be admitted into the corporation of surgeons.^52^ As recalled by his biographer, Herbert Hutchinson, however, Hutchinson was critical of the way vivas were conducted at the Royal College of Surgeons. ‘He sought to eliminate the personal element’ and ‘felt very keenly the unfairness of the viva-voce’. ‘The state of health and temper of the examiner, the difference or similarity of temperament between examiner and examined, might count for much; and make or mar the future of a young man.’^53^ Horsley too was also known for his ‘contempt for examinations as any real test of knowledge or capacity’.^54^ In *The Viva*, the vignette at the front table of the examiner who is becoming exhausted and exasperated with the student offers a cautionary lesson about the dangers of the viva voce.

The portraits and busts in Brooks’ *The Viva* represent bodily fragments, much like the specimens in jars, the surgeons’ bodies having been similarly anatomized. Furthermore, in the portrait of Carlisle, we do not see his head but we do see a skull, another body part, which relates to the skull in the painting’s foreground. More generally, the proliferation of body parts in the picture betrays an underlying anxiety about the practice of dissection. Aside from the surgeons and surgical students, the only complete bodies that appear in *The Viva* are those of the classical statuettes – art having managed to return the body to an ideal wholeness.

In fact, it is precisely this tension between the part and the whole, or generalized anatomy and individual identity, which forms the basis of the plot of Thompson’s *Charley Kingston’s Aunt*. Charley recalls that ‘a body designated for anatomical dissection … is divided for the purpose into certain definite “parts”’, and that ‘[t]he “part” which I required was the head and neck’.^55^ ‘Uncovering the bust for general inspection, I remarked the unusually pale and emaciated conditions of the subject … Then I commenced my work.’^56^ Charley is beset by the uncanny sensation that ‘the part’ is somehow familiar, but ‘I myself have always been a prey to that curious, semi-artistic facility for seeing resemblances, everywhere, in all bodies animate and inanimate, to other known forms and features’.^57^ Note how in the context of the dissecting room the ‘semi-artistic facility’ for identifying resemblances is invoked. Later that evening, having finally recognized the individual from the body part that he had been dissecting, he proclaims: ‘Good heavens! The old aunt!’^58^

## Painting surgeons: *An Octave for Mr Ernest Hart at Sir Henry Thompson’s House*

**Figure 6:**
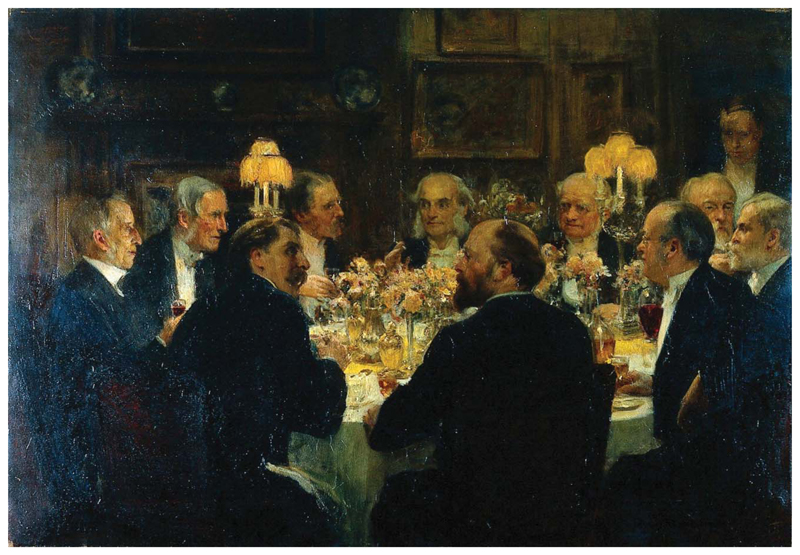
Solomon Joseph Solomon, *An Octave for Mr Ernest Hart at Sir Henry Thompson’s House*, *c*. 1897, oil on canvas, 71.5 x 103.5 cm. Wellcome Library, London. Clockwise: Ernest Abraham Hart, Thomas Spencer Wells, Joseph Fayrer, Thomas Lauder Brunton, W.H. Broadbent, George Anderson Critchett, Victor Horsley, Richard Quain, James Paget, Henry Thompson.

The two group portraits by Brooks that I have discussed thus far can convincingly be described as medical group portraits by virtue of the fact that they feature medical men situated within the medical institution of the Royal College of Surgeons. While neither painting shows them performing actual surgeries, they do show surgeons at work: attending a council meeting and administering an examination. By contrast, Solomon Joseph Solomon’s *An Octave* (Figure 6) shows surgeons as leisured gentlemen enjoying a dinner party.

*An Octave* is a study in impressionistic light effects. The surface is thick with paint and there are visible smudges, especially in the artist’s treatment of the flowers and lamps. A striking visual effect is also produced by the shine on the silver and glassware. Solomon was a Royal Academician but was also involved in the formation of the New English Art Club. Although he is usually associated with the production of academic history paintings in the style of Frederic Leighton and Alexandre Cabanel, such as *Samson* (1884) and *Ajax and Cassandra* (1886), *An Octave* is illustrative of Solomon’s work as a portrait painter.^59^

Solomon’s painting features well-known medical men from the late nineteenth century – (clockwise from centre back) Ernest Abraham Hart, Wells, Joseph Fayrer, Thomas Lauder Brunton, William Broadbent, George Anderson Critchett, Horsley, Richard Quain, Paget and Thompson – assembled around an opulently adorned dining table. They all wear black dinner suits and are engaged in leisured activities, indicating their status as gentlemen of the upper middle class. There is nothing in the scene to suggest that they are surgeons. Rather, the painting fits within a body of work by Solomon of fashionable members of society at leisure, including his 1884 painting *Conversation Piece*. These works are characterized by their attention to the rendering of light and shadow, executed through the use of loose, visible brushwork.

The title, *An Octave for Mr Ernest Hart at Sir Henry Thompson’s House*, informs us that the painting is of one of Thompson’s famous octave dinner parties of eight men of different professions at which eight dishes were served. These dinner parties reinforced a homosocial network of upper-middle-class men; in Solomon’s painting the working-class butler is compositionally excluded from the circle, and women were never invited. In Solomon’s scene there are, in fact, more than eight men dining and they are all members of the same profession. Consequently, the painting starts to appear more like a group portrait than a genre scene or conversation piece, the domestic interior becoming a site for professional networking.^60^

The letters and diaries of the sitters reveal the close relationships that existed between them. Broadbent, physician, expert neurologist and authority on heart disease, wrote that ‘I have always looked up to and reverenced Sir James Paget’.^61^ Horsley travelled to Paris with Brunton, the authority on the action of drugs on the heart. Additionally, it is likely that Thompson, as professor of clinical surgery and then consulting surgeon at University College Hospital, taught Horsley when he was a student there from 1874 to 1880. Horsley subsequently became house surgeon to John Marshall and surgical registrar at UCH.^62^ In Solomon’s painting, Horsley and Thompson, who appear one above the other, are visually connected through their similarly cocked and turned heads, and through their handlebar moustaches, giving the impression that the younger Horsley has been positioned as successor to the more senior Thompson.

Preparatory painted sketches of each individual figure indicate that the sitters were strategically assembled, not unlike Brooks’ group portrait of the councillors. In other words, the arrangement of the figures was not haphazard. Hart – surgeon, medical journalist and guest of honour at Thompson’s banquet – is situated in the centre of the composition, visible through the gap between Critchett and Horsley, his face the most clearly visible in the group. Thompson and Wells are positioned on either side of Hart. While both were celebrity doctors in their day, there is further significance to this compositional arrangement. Thompson was the foremost advocate for cremation during the second half of the nineteenth century and, significantly, artists and doctors were particularly well represented among its supporters, especially Hart and Wells. The highly publicized project of hygiene reform counteracted the popular perception of surgeons as bloody carpenters, with cremation offering an arguably better fate for a corpse than dissection.

Their arguments in favour of cremation were generally that decomposing corpses were unhygienic, even toxic, particularly in expanding urban centres with growing populations, and that cremation was a cleaner and healthier way to dispose of the dead.^63^ In fact, Wells read a paper at the British Medical Association meeting in Cambridge in 1880 in which he cited many arguments in favour of cremation with the aim of enlisting the support of the medical profession. The outcome was 120 signatures for a letter to the Home Secretary appealing for new legislation.^64^ Correspondence between Hart and Thompson held in the Wellcome Library refers to a common endeavour, possibly a shrouded reference to cremation. Thompson wrote to Hart that ‘I have always consulted you mainly in all this matter, because you were the only man who knows what to do’, and later that ‘Wells has written me that he is having some communication with Lord Doiley who “might be” induced to bring in a bill’.^65^ Therefore, the painting, through the arrangement of the figures, celebrated a community of medical men and highlighted those individuals who participated in significant contemporaneous debates around hygiene reform.^66^

Having established that Solomon’s painting is a medical group portrait, what is especially significant is that the setting is the ‘aestheticized’ interior of Thompson’s home. In *Celebrities at Home* of 1878, Edmund Yates described the inside of Thompson’s house on Wimpole Street, taking particular note of his fine and decorative art collection: ‘Sir Henry Thompson’s two consulting-rooms are full of china … the brilliant blue and white contrasts effectively with the dark green of a bower of palms and ferns, and the rich warm tone of several fine Ettys. The atmosphere of art pervades the entire house.’^67^ In the background of *An Octave* one can identify Thompson’s art and porcelain collection. Porcelain collecting was closely associated with the ‘art for art’s sake’ movement by this time, with Thompson having acquired pieces of blue and white china from artists James McNeill Whistler and Dante Gabriel Rossetti. Additionally, Thompson illustrated a book about porcelain with Whistler in 1878, further connecting the surgeon to the artistic circles of the late nineteenth century.^68^

By collecting art, doctors fashioned themselves into cultured gentlemen, connoisseurs with good taste. In *Charley Kingston’s Aunt*, Thompson commented on the practice of art collecting among doctors: ‘it is an indisputable [fact], that almost every medical man of ordinary intelligence, who achieves a fair share of success in his profession … becomes a fine art collector of some sort’. He continued: ‘In the towns and in the metropolis he collects engravings, etchings, watercolour drawings, or he may possess works in oil by the best artists, not merely of modern time, but here and there a choice old master. Or he treasures up old plate, or Wedgwood, or Sèvres, or Oriental china, or ivories, or woodcarvings.’^69^

As well as collecting art Thompson was an amateur artist, exhibiting paintings on occasion at the Royal Academy and Paris Salon. This was the subject of satirical treatment in *Punch’s* ‘Fancy Portrait’ (1881, Figure 7), in which Thompson is referred to as ‘Great Artist-Surgeon, who so well is able, To Point a Pencil and Adorn a Table?’ The reference to Thompson’s skill at adorning a table and the inclusion in the picture of a ‘menu for dinner’ references his famous octaves and his publication *Food and Feeding* (1880).^70^ Yet, in the caricature the table is not the elaborately adorned dinner table of Solomon’s painting; it is the wooden dissecting table with what looks like carpentry tools. Moreover, in the background there is a lay figure, which was used as an aid by artists, and a skeleton, which hovers as a macabre reminder of the dead bodies with which the ‘great artist-surgeon’ works. The caricaturist draws on the old stereotype of surgeons as manual labourers whose business is the violent cutting open of living and dead bodies.

Historically artists and surgeons collaborated in the study of anatomy, and this continued throughout the nineteenth century in England. But there emerged in the century’s final decades a social network of gentleman artists and surgeons, in which Thompson was a central figure, and who were more likely to be photographed and painted dining together than dissecting together. Thompson numbered among his friends several influential members of the late Victorian art world, including Lawrence Alma-Tadema and John Everett Millais, and together they enjoyed leisured pursuits befitting gentlemen of the upper middle class.

**Figure 8:**
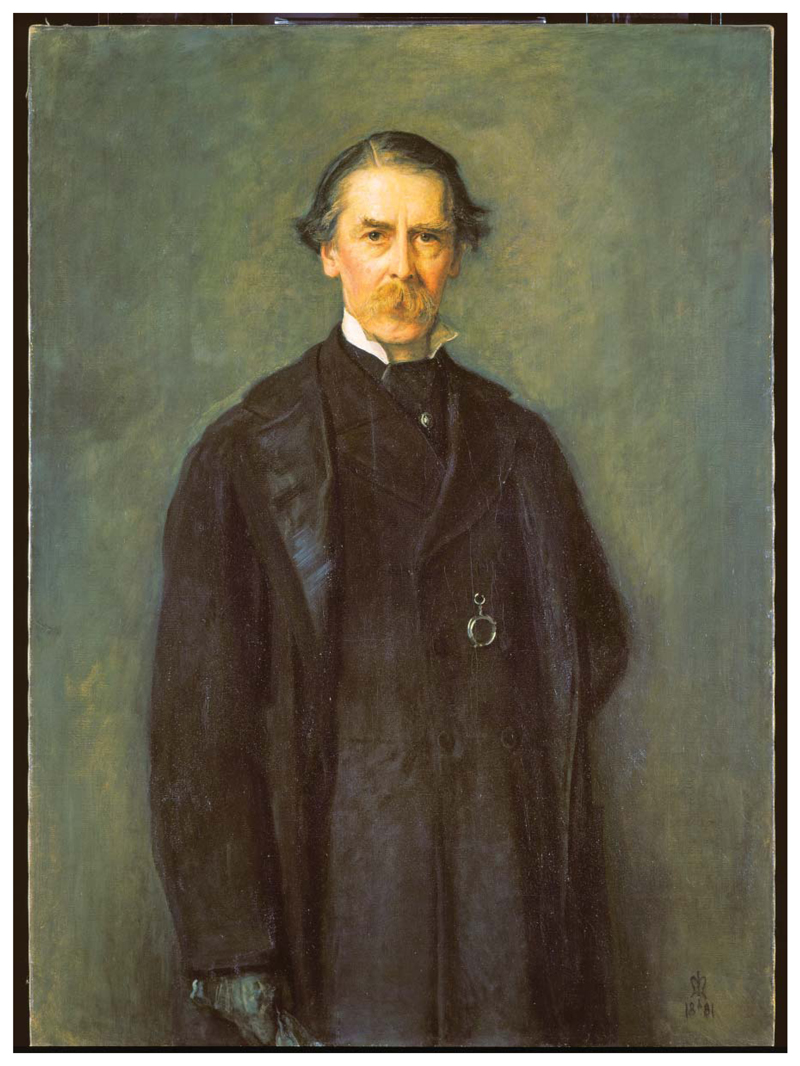
John Everett Millais, *Sir Henry Thompson, Bt*, 1881, oil on canvas, 125.7 x 91.4 cm. © Tate, London 2012.

Millais exhibited his famous portrait of Thompson at the Royal Academy in 1882 (Figure 8), but what is little known is that during a weekend away at Robert Collier’s house, Thompson painted Millais’ portrait.^71^ The following was recounted by Thompson to Millais’ son, John Guille Millais, and published in *The Life and Letters of Sir John Everett* Millais (1899), along with reproductions of Thompson’s portrait of Millais and Millais’ portrait of Thompson:

Sir Robert Collier (afterwards Lord Monkswell) invited Sir Henry James (now Lord James), Sir John Millais, and myself to his house in Essex, from Saturday to Monday, as a whist party. It was cold, wintry weather, near Christmas, and on Sunday morning James, being then Attorney-General, and Collier, being Lord Justice of Privy Council, thought that officially they had better go to church. Millais and I elected to stay at home. Said I to Millais, ‘What are you going to do?’ He said ‘Sit here and smoke’. ‘Then,’ said I, ‘I will get a little mill-board, set a palette and paint you, if I may’. ‘All right,’ said he. He sat quite still with his back to the window, and I set to work. I worked at my study the best part of two hours, and put it on the mantelpiece. Soon afterwards the two others came in. ‘There,’ said Millais, ‘that’s what *we’ve* been doing!’ It was never touched afterwards, and they all thought it would do as it was.^72^

This incident illustrates that portraits not only inscribed elevated social status, they also sometimes constituted tokens of intimate friendship between artists and surgeons.^73^

Solomon and Thompson, it appears, moved in the same social circles and knew each other beyond the relationship of portraitist and sitter.^74^ But perhaps the explanation for the choice of Solomon as the artist for *An Octave*, or conversely Solomon’s choice of subject, lies in the figure of Hart. Both Solomon and Hart were high-profile members of the Victorian Anglo-Jewish community, and, significantly, Solomon featured Hart in an earlier work entitled *Your Health*, which was exhibited at the Royal Academy in 1894. Therefore, in two major works, Solomon honoured Hart, a Jewish surgeon who represented a new generation of cosmopolitan Jewish professionals.^75^

**Figure 9:**
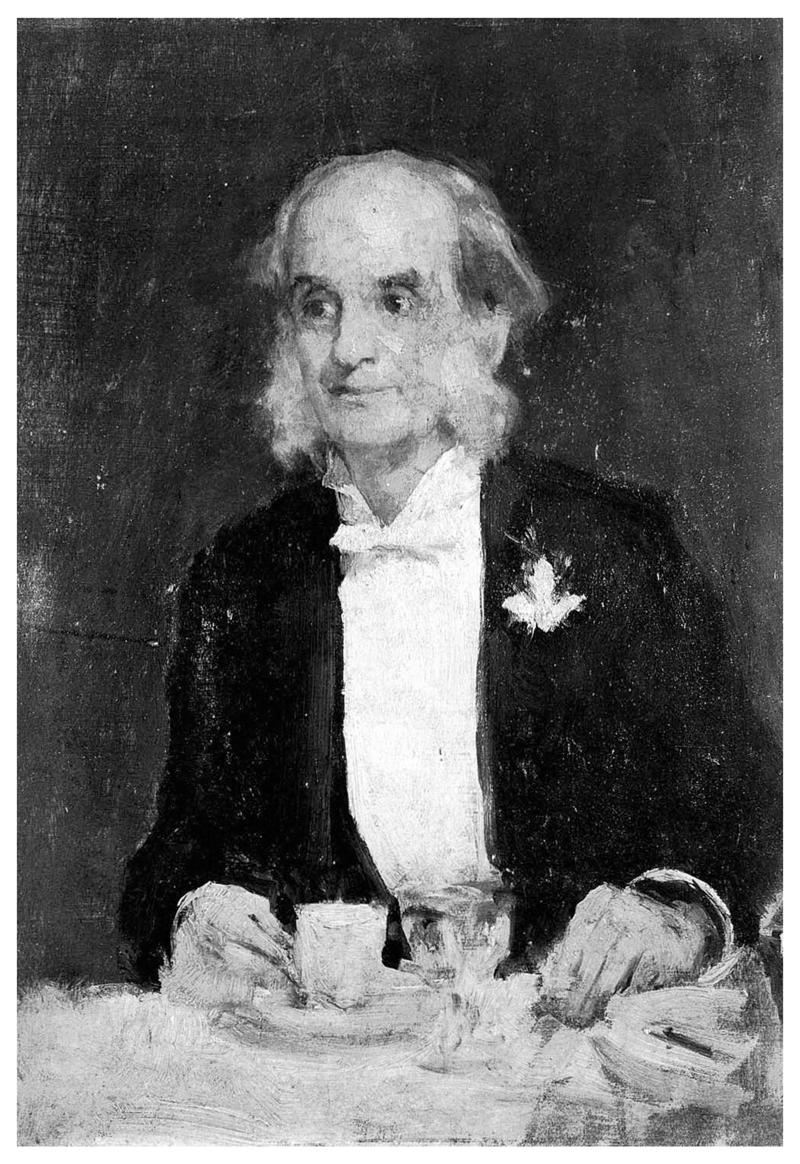
Solomon Joseph Solomon, Study of Ernest Abraham Hart, oil on wood, 35.5 x 26 cm. Wellcome Library, London.

What is so provocative about Solomon’s depiction of Hart, however, is that he is shown smoking a cigarette. According to Yates, Thompson also smoked cigarettes, his afternoon ritual consisting of a ‘slight snack followed by a cigarette’.^76^ A consideration of the individual painted studies for *An Octave* in relation to the finished canvas reveals changes that were made to the final composition, including having Hart smoking a cigarette instead of sipping tea (Figure 9). This is a remarkable inclusion considering that, while smoking cigars and pipes was part of the rituals of upper and middle-class male leisure, the cigarette, which was only widely available in England from the 1880s onwards, was associated with debased mass production, dandies, avant-garde artists and the gender transgressions of the New Woman.^77^

The cigarette was widely considered to be inferior to the pipe and cigar, and those men who smoked it were branded not ‘man enough’ to smoke properly.^78^ In an engraving of a medical student by John Orrin Smith, after Joseph Kenny Meadows, published in *Heads of the people; or, Portraits of the English* (1840) the morbid pleasures that the medical student takes in murder and dissection are tied up with his less-than-manly demeanour and his habit of smoking.^79^ After all, smoking in dissecting theatres was a common way for medical students to mask the stench of rotting cadavers.^80^ *Charley Kingston’s Aunt* makes repeated mention of tobacco smoking among medical students, especially the smoking of cigars. In one instance Charley’s friend, the house surgeon, Allison, settles into an armchair with a ‘cigarette in his mouth’.^81^

With reference to other painted male cigarette smokers from the final decades of the nineteenth century, the depiction of Hart with a cigarette may be understood as an attempt at forging an alternative yet still acceptable late-Victorian masculine identity. The artist James Tissot produced two portraits of well-known Victorian men smoking: *Colonel Frederick Gustave Burnaby* (1870, Figure 10) smoking a cigarette and the art collector *Algernon Moses Marsden* (1877) smoking a cigar. Both men are depicted as debonair, languid, cultured gentlemen in domestic spaces filled with objects that connote their exploits, including porcelain collecting in the case of Marsden.

Colonel Burnaby is shown amid items that reflect his occupation: the shiny silver breastplate and helmet, books and a world map, all signalling the figure’s military successes and foreign travel. Yet, despite Colonel Burnaby’s military achievements, he is depicted as languid and even effete, and the cigarette contributes to this. He elegantly holds his cigarette so that it is positioned in the centre of the composition. Its long slender appearance is mirrored in Burnaby’s elongated legs, and its white colour picked up in the white sash across his chest and the horizontal line formed by the base of the map’s frame. Taking this a step further, Tissot’s portrait of Burnaby is rendered predominantly in white, red and black, precisely the colours of the cigarette itself, and this recalls the cigarette colour scheme of the 1894 *New Woman* poster by Albert George Morrow for the Comedy Theatre in the Victoria and Albert Collection. If the cigarette in the bottom corner of Morrow’s poster signifies the New Woman, then Colonel Burnaby with his cigarette represents a new kind of man.

Returning to Solomon’s depiction of Hart, his appearance amid Thompson’s art and ceramic collection, along with his casual smoking of a cigarette, indicates that he represents, not just a new type of modern, Victorian man, but a new type of modern, Jewish, ‘aestheticized’ Victorian surgeon. Christopher Lawrence offers a possible explanation for the adoption by surgeons of ‘qualities usually specified as feminine’ when he notes that ‘they could … be invoked to counter the ascribed coarseness of surgeons’.^82^ But, more than that, the gentleman artist-surgeon, depicted by Solomon in *An Octave*, signalled a departure from earlier nineteenth-century models of the surgeon as institutionalized authority and middle-class professional (as per Brooks’ *Council of the College*) and scientist (as per *The Viva*).

## Conclusion

When the critic for *The Times* reviewed Millais’ portrait of Thompson, he compared George Frederick Watts’ and Millais’ styles of portrait painting, stating that ‘Mr Watts tries to show us what there is *in* a man … Mr Millais shows us what there is *out* of him. One opens the letter and the other reads the envelope.’^83^ Indeed, Victorian critics were eager to identify in Millais’ rendering of Thompson those indicators of the sitter’s ‘strong individual character’ and his ‘look of keen precision’, which were characteristic of ‘the great surgeon’.^84^ Millais invited his viewers to read intellect in the great surgeon’s tall forehead, concentration in his direct stare and the creases in his brow, distinction in his straight nose, moderation in his stark black dinner suit and an attention to detail in his monocle. Thompson’s envelope was that of the consummate Victorian gentleman.

British portraits of surgeons from the 1880s and ’90s consistently showed them as suited gentlemen. In the words of James Ewing Mears in *The Evolution of Surgery* (1904), the surgeon had climbed from the position of ‘bleeder and barber’ to ‘pathologist [and] diagnostician’ and was now ‘crowned above all as the refined and cultured gentleman’.^85^ Group portraits, such as those by Brooks, asserted the professionalism of surgery, the increased power of medical corporations and the difficulty in gaining admission to the profession. Other portraits, such as Solomon’s *An Octave*, celebrated the emergence of a new type of gentleman artist-surgeon who dabbled in art, collected porcelain, smoked cigarettes, decorated his house and held lavish dinner parties. But as the ‘Fancy Portrait’ of Thompson from *Punch* attests, the gentleman artist-surgeon was not an unproblematic category, for in the ungainly nature of the oversized surgical knife-turned-pencil, and in the mess of saws, scissors and screws, the association of surgery with butchery could not be so easily concealed beneath a tailored dinner suit.

## Figures and Tables

**Figure 1:**
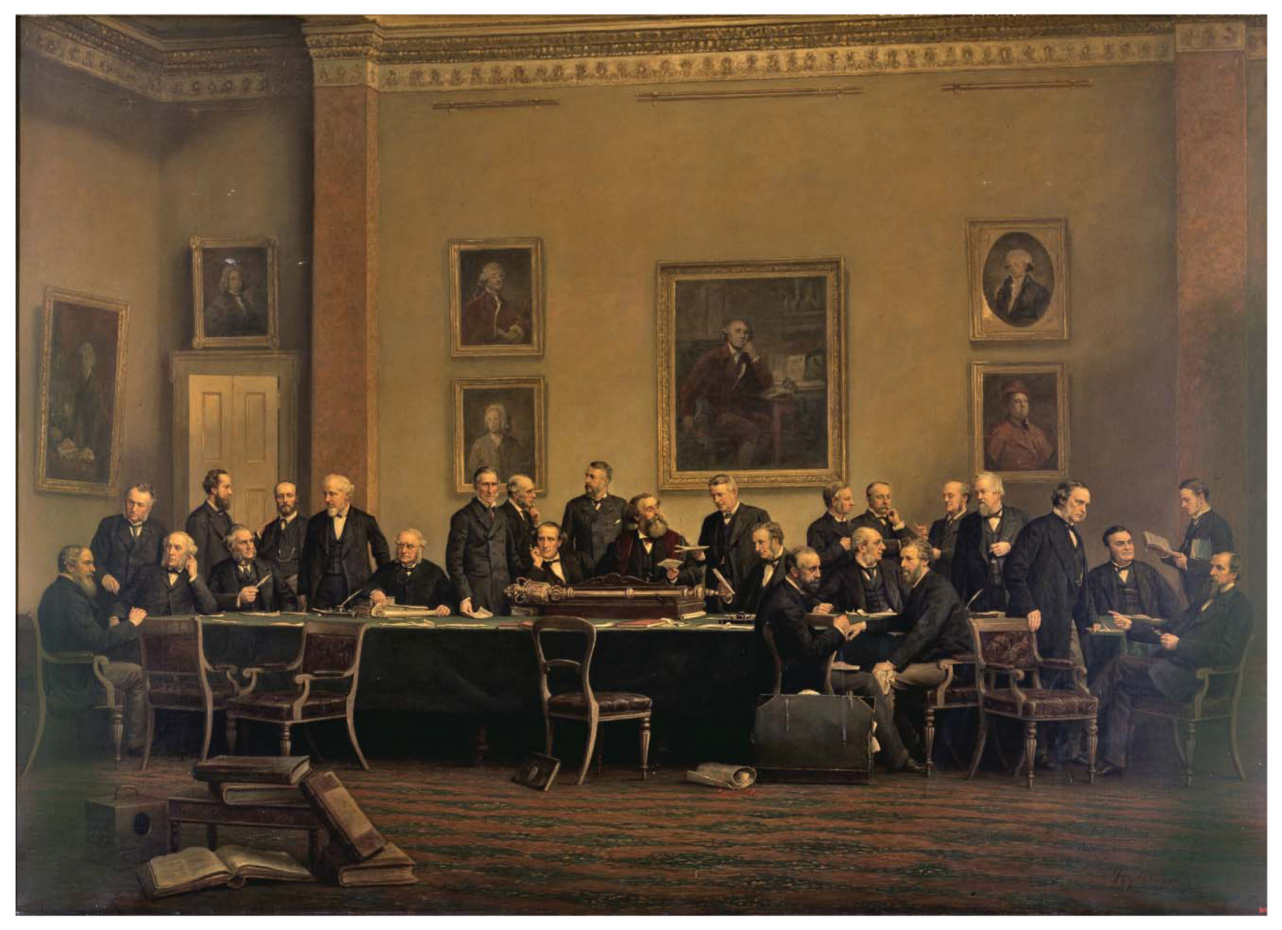
Henry Jamyn Brooks, *Council of the College, 1884–85*, 1887, oil on canvas, 151 x 212 cm. Courtesy of the Royal College of Surgeons of England. Left to right: John Whitaker Hulke, Thomas Bryant, William Cadge, Arthur Edward Durham, Henry Power, George Lawson, Edward Lund, Thomas Spencer Wells, James Paget, John Croft, William Scovell Savory (Vice President), William MacCormac, John Cooper Forster (President), Edward Trimmer (Secretary), Timothy Holmes (Vice-President), Christopher Heath, Matthew Berkeley Hill, John Eric Erichsen, William Allingham, Thomas Smith, Sydney Jones, John Marshall, Joseph Lister, John Wood, Frederic Greville Hallett (Assistant Secretary), Jonathan Hutchinson.

**Figure 5:**
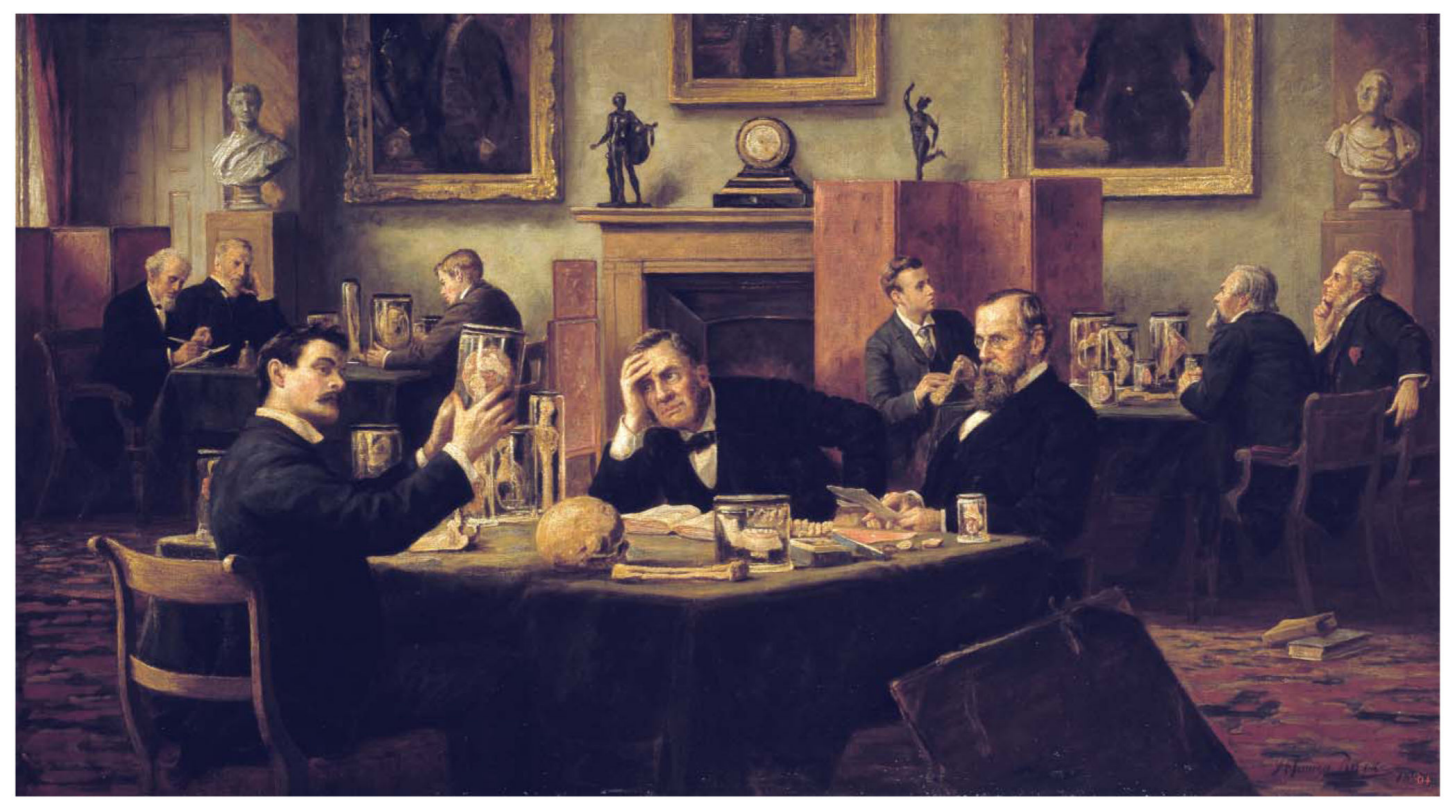
Henry Jamyn Brooks, *The Viva*, 1894, oil on canvas, 77 x 141 cm. Courtesy of the Royal College of Surgeons of England. Left to right: Edward Lund, William MacCormac, John Wood, Jonathan Hutchinson, John Marshall, Frederick Le Gros Clark.

**Figure 7:**
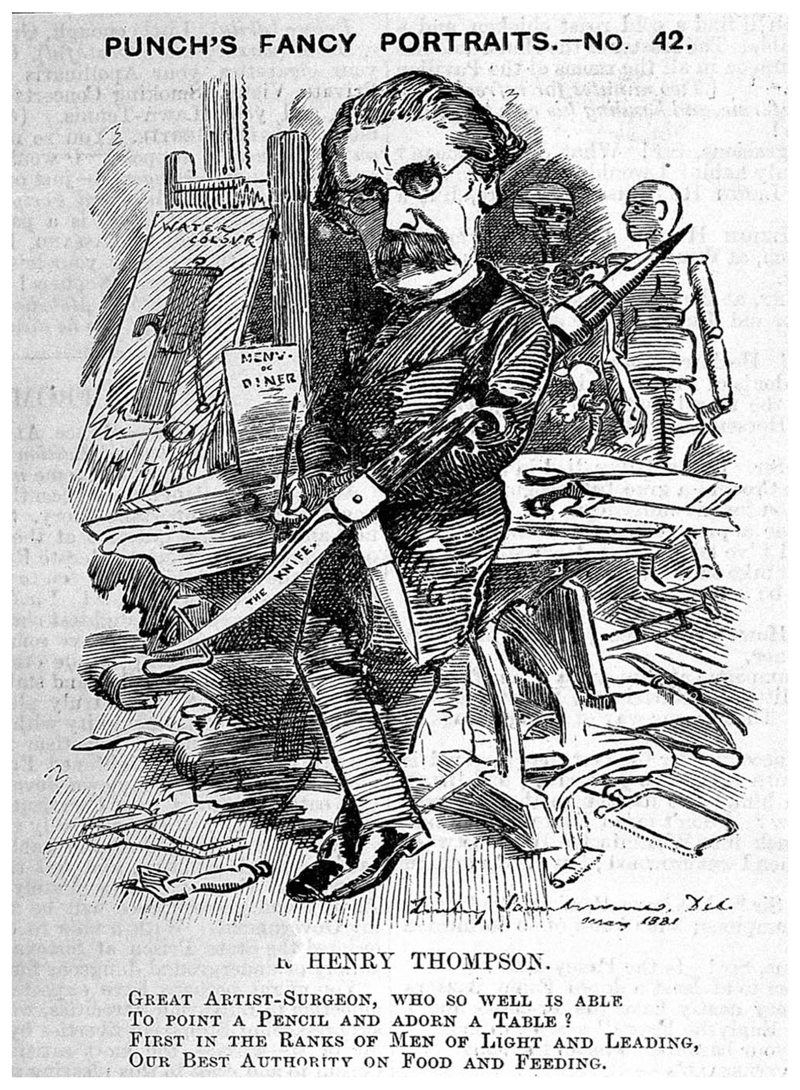
Lindsay Sambourne, *Punch’s Fancy Portraits – No. 42. Dr. Henry Thompson*, *Punch* 81 (1881), 46. Wellcome Library, London.

**Figure 10:**
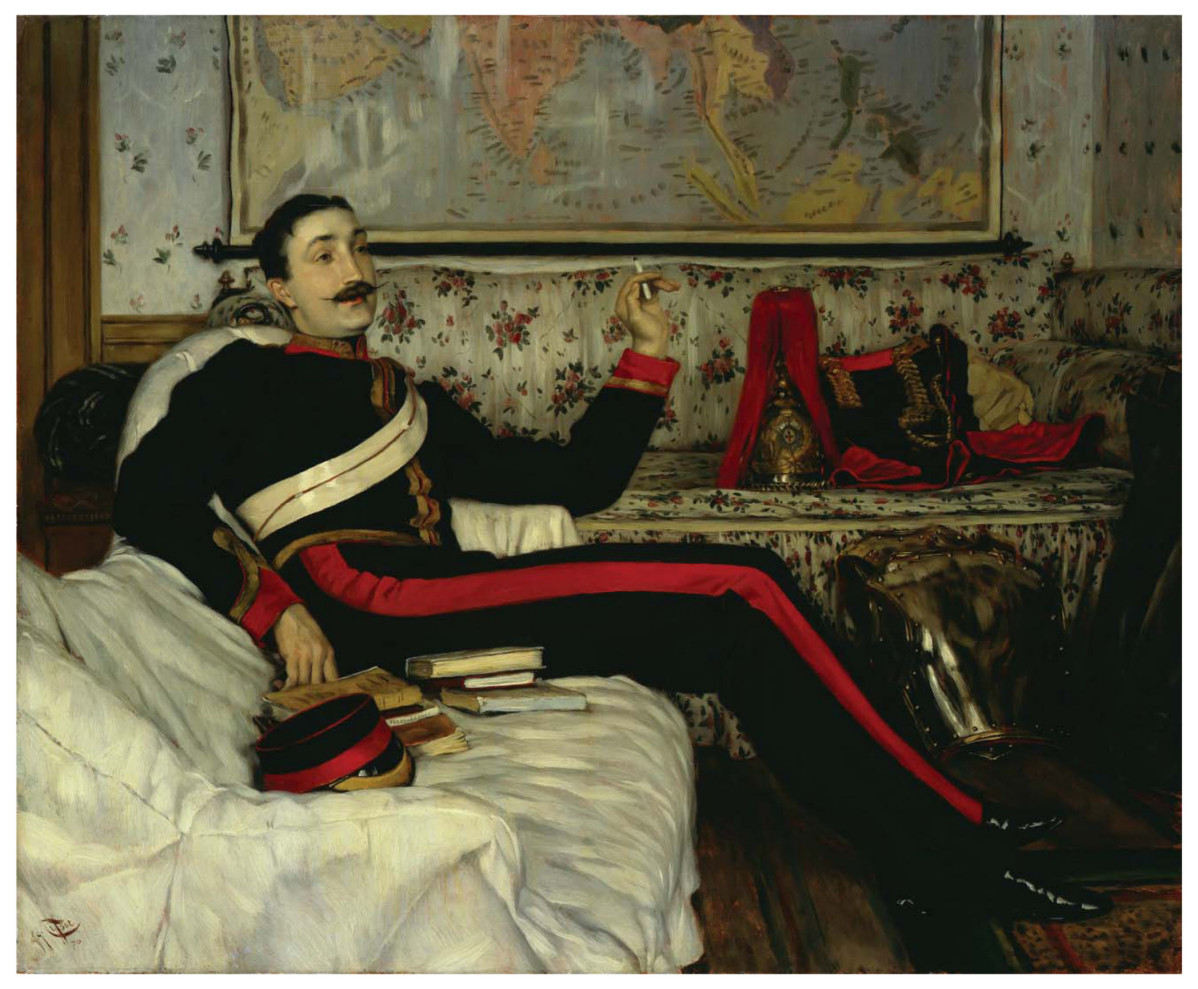
James Tissot, *Frederick Gustavus Burnaby,* 1870, oil on panel, 49.5 x 59.7 cm. © National Portrait Gallery, London.

## Notes

1 Thompson [Pen Oliver], *Charley Kingston’s Aunt*, 15.

2 Perkins, *The Rise of Professional Society*.

3 For example, in *The Victorians and the Visual Imagination*, Flint considers the pursuit of visibility in the medical sciences as expressed through Victorian literature, especially the novels of George Eliot. In ‘Photography as Witness, Detective, and Imposter’, Jennifer Tucker interrogates the use of photography as scientific evidence. In ‘The Image of Objectivity’, Daston and Galison offer an account of the shift during the nineteenth century from idealism to ‘objective’ naturalism in anatomical illustrations through a study of scientific atlases. These scholars and others address the complex issue of Victorian anxieties around error and misrecognition based on visual disturbances, deceptive appearances and subjective interpretations, which will support my observations about the inadequacy of vision to convey adequate information about the anatomical specimens pictured in Brooks’ *The Viva*.

4 Jordanova, *Defining Features*.

5 Richardson, *Death, Dissection and the Destitute*.

6 These include: Adams, *Dandies and Desert Saints*; Stephenson, ‘Leighton and the Shifting Repertoires of “Masculine” Artistic Identity in the Late Victorian Period’ and ‘Precarious Poses’; Edwards, ‘A Portrait of the Artist as a Young Aesthete’ and *Alfred Gilbert’s Aestheticism*; Kim, *Painted Men in Britain, 1868–1918.*

7 Maugham, *Of Human Bondage*, 148.

8 Riegl, *The Group Portraiture of Holland*, 194.

9 The controversy surrounding *The Gross Clinic* is discussed by Henricks in ‘Thomas Eakins’s Gross Clinic’ and Doyle in ‘Sex, Scandal, and Thomas Eakins’s *The Gross Clinic*’. Mention should also be made of John Harley Warner’s study of photographs of American medical students in dissecting rooms from the late nineteenth and early twentieth centuries, photographs, which he classifies as group portraits. Warner, ‘Witnessing Dissection’.

10 Hunter, ‘The Sleep of Reason’, 49.

11 11 Ibid., 56.

12 Kim, *Painted Men in Britain*, 84.

13 By the end of the century Brooks had established a reputation for producing group portraits of different corporations, but at the time of his painting *Private View* he was little known among the art establishment. Philip Calderon (Keeper of the Royal Academy 1887–98) wrote to George Scharf (Director of the National Portrait Gallery): ‘I know very little of Mr Brooks’. Letter dated 18 October 1888, National Portrait Gallery, London, RP 1833b.

14 In her article ‘Painting Indoors’, Prettejohn discusses the ways in which Frederic Leighton, President of the Royal Academy from 1878 to 1896, who appears at the centre of Brooks’ *Private View*, offered evidence of his laborious academic working method while, at the same time, constructing himself as a gentlemanly connoisseur through photographic portraits.

15 National Portrait Gallery, London, RP 1833.

16 Thanks to Ludmilla Jordanova for alerting me to this connection.

17 Jordanova writes that ‘Walker’s print is remarkable for its depiction of the scientists of a particular country as a collectivity. The print, of course, embodies a strategic fiction. It is not a genuine group portrait, but an invented scene based on pre-existing individual portraits. The men portrayed never met as a group, and it is possible that some of them never even set foot in the Royal Institution in Albemarle Street, London. Yet the image makes strong claims to authenticity … Through this invented scene the image evokes the intellectual achievements of Great Britain half a century earlier.’ Jordanova, ‘Science and Nationhood’, 193.

18 Christopher Lawrence observes that ‘it is a striking feature of the representation of surgeons in the late eighteenth and nineteenth centuries that they are rarely depicted with their surgical instruments, that is, the tools of their trade. They are, however, frequently represented as men of science and learning’. Lawrence, ‘Medical Minds, Surgical Bodies’, 188.

19 For an extended discussion of the claims of nineteenth-century doctors to different forms of knowledge, especially scientific knowledge, see Shortt, ‘Physicians, Science, and Status’.

20 Jordanova, *Defining Features*, 41.

21 Sarafianos, ‘The Natural History of Man and the Politics of Medical Portraiture in Manchester’, 105.

22 ‘Sir T. Spencer Wells, Bart., on behalf of the Subscribers, offered for acceptance of the College the picture by Mr.H. J. Brooks of the Council in 1884–5. Resolved: That the picture be accepted with thanks. Royal College of Surgeons of England, Extraordinary Meeting of the Council on Thursday 5th May, 1887’. *Minutes of Council*, *1885–87*, 265.

23 Harvey, *Men in Black*, 195.

24 Cope, *The Royal College of Surgeons of England: A History*, 2; Corfield, *Power and the Professions in Britain 1700–1850*, 154.

25 Cope, *The Royal College of Surgeons of England*, 108.

26 Ibid., 109.

27 Ibid.

28 Ibid., 111.

29 Entries for John Marshall and John Wood in the *Oxford Dictionary of National Biography*, http://www.oxforddnb.com/view/article/18145?docPos=8 and http://www.oxforddnb.com/view/article/29882?docPos=11 (accessed January 21, 2013).

30 The title is listed as *The Viva* on the Royal College of Surgeons’ database. We have no evidence, however, that that title was given to the work by the artist or sitters.

31 In 1858, the passage of the Medical Act ‘ratified the pluralist system, whereby either the corporations or the universities could examine and certify a candidate’s fitness to practice medicine’. Bynum, *Science and the Practice of Medicine*, 179. Also see Roberts, ‘The Politics of Professionalization’.

32 ‘The picture is not of giving an Anatomy Lecture but represents J.H and John Wood examining an unfortunate student. It recalls the mental horrors of that examination to me even now over 60 years ago.’ Letter from Roger Jackson Hutchinson (son of Jonathan Hutchinson) to Dr Sharp, dated January 13, 1948, Royal College of Surgeons Collection.

33 Bynum, *Science and the Practice of Medicine*, 179.

34 For more on this see Newman, *The Evolution of Medical Education in the Nineteenth Century*, 202–7.

35 Thompson, *Charley Kingston’s Aunt*, 180–1.

36 Ibid., 182.

37 Marshall, *A Description of the Human Body*.

38 Hutchinson initially established a museum in 1888 in his house at Inval. Construction of the museum in Haslemere was completed in 1894. The painting seems to have been housed at Hutchinson’s educational museum at Selby until 1939 when the Hutchinson family presented it to the Haslemere Museum. “‘Observables” at the Royal College of Surgeons’, *Annals of the Royal College of Surgeons of England*, 285. Swanton, *A Country Museum*, 73. Roger Hutchinson presented *The Viva* to the Royal College of Surgeons in 1948. It was hung in the Refectory at the College ‘where post-graduate students can get a preview of the setting for the Viva Voce Examination’. “‘Observables” at the Royal College of Surgeons’, 286.

39 The production of the painting was contemporaneous with the opening of the Haslemere museum.

40 Swanton, *A Country Museum*, 7.

41 Ibid., 37.

42 Quoted in ibid., 23.

43 Entry for 1906 in Swanton’s *A Country Museum* pamphlet (London: Coracle, 1992), extracted from *A Country Museum*, which describes Hutchinson’s article entitled ‘Museums and Museums’ published in *The Haslemere Museum Gazette*.

44 Quoted in Swanton, *A Country Museum*, 36.

45 See photographs of Horsley in the Wellcome Library (12927i). In his biography of Horsley, Stephen Paget comments on his outspokenness, which alienated older members of the medical profession. *Sir Victor Horsley*, 275–79. Paget writes: ‘[S]ome of us found offence where none was intended; we took seriously his light use of flamboyant words, which were nothing more than the stand [*sic*] of his early years; mocking heroic epithets, to be let off like fireworks’. He continues: ‘The fact remains, that he alienated men whom he could just as easily have bound to himself in friendship’. Paget, *Sir Victor Horsley*, 277.

46 Entry for Victor Horsley in the *Oxford Dictionary of National Biography*, http://www.oxforddnb.com/view/article/34000 (accessed January 21, 2013). Horsley passed his final examination for the membership of the Royal College of Surgeons in November 1880. Paget, *Sir Victor Horsley*, 36.

47 In *Artistic Anatomy*, Mathias Duval featured a sketch of the ‘Facial Angle of an Antique Head (Apollo Belvedere)’ as representative of the most highly evolved physical type, 176.

48 I am concerned here with the relationship between medical diagnostic looking and touching, and artistic production and evaluation. Historians of medicine have written about the innovations in medical diagnostics that took place during the nineteenth century through the deployment of the senses of touch and hearing, and the development of new technologies, such as the stethoscope. These include Lachmund, ‘Between Scrutiny and Treatment’; Sturdy, ‘Making Sense in the Pathology Museum’.

49 Thanks to Sam Alberti for identifying these portraits.

50 Lawrence, “‘Democratic, Divine and Heroic”’, 7

51 Swanton, *A Country Museum*, 18.

52 Perkins states that ‘[t]he professional ideal was based on trained expertise and selection by merit, a selection made not by the open market but by the judgement of similarly educated experts’. *The Rise of Professional Society*, xxii.

53 Hutchinson, *Jonathan Hutchinson*, 195–6. In October 1895, Hutchinson took up the point of the examination system in an address he delivered in Liverpool entitled ‘On Examinations as an Aid to Education’, given at the opening of the Medical College. Hutchinson, *Jonathan Hutchinson*, 195.

54 This was written by one of Horsley’s contemporaries, J. E. Hine, and published in Paget, *Sir Victor Horsley*, 29.

55 Thompson, *Charley Kingston’s Aunt*, 14–15.

56 Ibid., 34.

57 Ibid.

58 Ibid., 36.

59 *Solomon J Solomon RA*, exhibition catalogue.

60 This is corroborated by the fact that Solomon seems to have appropriated the composition of D. George Thompson’s *A Literary Party at Sir Joshua Reynolds’s*, 1851, published by Owen Bailey, after James William Edmund Doyle (National Portrait Gallery, London, D14518). My thanks to Brian Hurwitz for bringing this to my attention.

61 Letter from Broadbent to ‘S,’ dated March 5, 1893, published in Broadbent, ed., *The Life of Sir William Broadbent*, 247.

62 Entry for Victor Horsley in the *Oxford Dictionary of National Biography*.

63 Hamlin, ‘Good and Intimate Filth’, 15–16.

64 Shepherd, *Spencer Wells,* 112.

65 Letter not dated, Wellcome Library, Western Ms. 5423, from the collection of George Grey Turner, FRCS (acc. 347628).

66 ‘Public health provided a particularly appropriate field in which a medical subgroup could advance its professional status in the name of science’. Shortt, ‘Physicians, Science and Status’, 65.

67 Yates, *Celebrities at Home*, vol. 2, 28–9.

68 *A Catalogue of Blue and White Nankin Porcelain Forming the Collection of Sir Henry Thompson*.

69 Thompson, *Charley Kingston’s Aunt*, 129–31.

70 Included in the cartoon is a canvas showing a water pump with the inscription ‘Water Colour’. As well as satirizing the relationship between the artistic medium of watercolour and the Victorian reform of the water and sewage systems, it may reference the colour of the urine samples that Thompson observed in his famous work on diseases of the urinary organs. Thompson, *Clinical Lectures on Diseases of the Urinary Organs*.

71 Robert Collier, a distinguished advocate and later judge, was also an amateur artist, exhibiting frequently at the Royal Academy and Grosvenor Gallery.

72 Millais, ed., *The Life and Letters of Sir John Everett Millais*, 144–7.

73 It was common for portraits to be exchanged as tokens of friendship between artists, such as among the members of the Pre-Raphaelite Brotherhood. What is significant about the exchange of portraits between Millais and Thompson is that one was an artist and the other a surgeon.

74 Thompson and Solomon are present in a photograph of a banquet at the Royal Academy in honour of Alma-Tadema. Dated 4 November 1899 in the National Portrait Gallery Collection, London (NPG x19022).

75 Finestein, *Jewish Society in Victorian England*, 154–5. For a further discussion of Jewish doctors in the late Victorian period, including Hart see Cooper, *Pride Verses Prejudice*, 11–30.

76 Yates, *Celebrities at Home*, vol. 2, 34.

77 Berman, ‘Edvard Munch’s Self-Portrait with Cigarette’.

78 Hilton, *Smoking in British Popular Culture 1800–2000*, 26–8.

79 Keir Waddington considers stereotypes of medical students in the early Victorian period and how these stereotypes changed over the course of the century in ‘Mayhem and Medical Students’.

80 Warner, ‘Witnessing Dissection’, 13.

81 Thompson, *Charley Kingston’s Aunt*, 160.

82 Lawrence, ‘Medical Minds, Surgical Bodies,’ 193.

83 Emphasis in original.

84 ‘The Royal Academy (First Notice)’, *The Times*, May 1, 1882. 4; ‘The Royal Academy’, *The Standard*, April 29, 1882, 2.

85 Mears, *The Evolution of Surgery*, 16.

## References

[R1] Adams James Eli (1995). Dandies and Desert Saints: Styles of Victorian Manhood.

[R2] Berman Patricia G (1993). Edvard Munch’s Self-Portrait with Cigarette: Smoking and the Bohemian Persona. The Art Bulletin.

[R3] Broadbent ME (1909). The Life of Sir William Broadbent.

[R4] Bynum William F (1994). Science and the Practice of Medicine.

[R5] (1878). A Catalogue of Blue and White Nankin Porcelain Forming the Collection of Sir Henry Thompson, illustrated by the autotype process from drawings by James Whistler, Esq., and Sir Henry Thompson.

[R6] Cooper John (2003). Pride versus Prejudice: Jewish Doctors and Lawyers in England, 1890–1990.

[R7] Cope Zachary (1959). The Royal College of Surgeons of England: A History.

[R8] Corfield Penelope (1995). Power and the Professions in Britain 1700–1850.

[R9] Daston Lorraine, Galison Peter (1992). The Image of Objectivity. Representations.

[R10] Doyle Jennifer (1999). Sex, Scandal, and Thomas Eakins’s *The Gross Clinic*. Representations.

[R11] Duval Mathias, Fenton Frederick E (1884). Artistic Anatomy.

[R12] Edwards Jason, Getsy David J (2004). A Portrait of the Artist as a Young Aesthete: Alfred Gilbert’s *Perseus Arming* (1882) and the Question of “Aesthetic” Sculpture in late-Victorian Britain. Sculpture and the Pursuit of a Modern Ideal in Britain, c.1880–1930.

[R13] Edwards Jason (2006). Alfred Gilbert’s Aestheticism: Gilbert amongst Whistler, Wilde, Leighton, Pater and Burne-Jones.

[R14] Finestein Israel (1993). Jewish Society in Victorian England.

[R15] Flint Kate (2000). The Victorians and the Visual Imagination.

[R16] Hamlin Christopher, Cohen William A, Johnson Ryan (2005). Good and Intimate Filth. Filth: Dirt, Disgust, and Modern Life.

[R17] Harvey John (1995). Men in Black.

[R18] Gordon Henricks (1969). Thomas Eakins’s *Gross Clinic*. The Art Bulletin.

[R19] Hilton Matthew (2000). Smoking in British Popular Culture 1800–2000.

[R20] Hunter Mary (2004–5). The Sleep of Reason: Art, Medicine and Sexuality in Henri Gervex’s *Avant l’Opération*. Object.

[R21] Hutchinson Herbert (1946). Jonathan Hutchinson: Life and Letters.

[R22] Jordanova Ludmilla, Cubitt Geoffrey (1998). Science and Nationhood: Cultures of Imagined Communities. Imagining Nations.

[R23] Jordanova Ludmilla (2000). Defining Features: Scientific and Medical Portraits 1660–2000.

[R24] Kim Jongwoo Jeremy (2012). Painted Men in Britain, 1868–1918: Royal Academicians and Masculinities.

[R25] Lachmund Jens (1998). Between Scrutiny and Treatment: Physical Diagnosis and the Restructuring of 19th Century Medical Practice. Sociology of Health and Illness.

[R26] Lawrence Christopher, Lawrence Christopher (1992). “Democratic, Divine and Heroic”: The History and Historiography of Surgery. Medical Theory, Surgical Practice: Studies in the History of Surgery.

[R27] Lawrence Christopher, Lawrence Christopher, Shapin Steven (1998). Medical Minds, Surgical Bodies: Corporeality and the Doctors. Science Incarnate: Historical Embodiments of Natural Knowledge.

[R28] Marshall John (1882). A Description of the Human Body: Its Structure and Functions Designed for the Use of Teachers in Schools and Young Men Destined for the Medical Profession, and for Popular Instruction Generally.

[R29] Maugham W Somerset (1991). Of Human Bondage.

[R30] Mears James Ewing (1904). The Evolution of Surgery.

[R31] Millais John Guille (1899). The Life and Letters of Sir John Everett Millais, President of the Royal Academy.

[R32] Newman Charles (1957). The Evolution of Medical Education in the Nineteenth Century.

[R33] Paget Stephen (1919). Sir Victor Horsley: A Study of His Life and Work.

[R34] Perkins Harold (1989). The Rise of Professional Society: England since 1880.

[R35] Prettejohn Elizabeth (1996). Painting Indoors: Leighton and his Studio. Apollo.

[R36] Richardson Ruth (2000). Death, Dissection and the Destitute.

[R37] Riegl Alois, Kain Evelyn M, Britt D (1999). The Group Portraiture of Holland.

[R38] Roberts Michael JD (2009). The Politics of Professionalization: MPs, Medical Men, and the 1858 Medical Act. Medical History.

[R39] Royal College of Surgeons of England (1887). Minutes of Council, 1885– 87.

[R40] Sarafianos Aris (2006). The Natural History of Man and the Politics of Medical Portraiture in Manchester. Art Bulletin.

[R41] Shepherd John A (1965). Spencer Wells: The Life and Work of a Victorian Surgeon.

[R42] Shortt SED (1983). Physicians, Science, and Status: Issues in the Professionalization of Anglo-American Medicine in the Nineteenth Century. Medical History.

[R43] Solomon J, Solomon RA (1990). exhibition catalogue.

[R44] Stephenson Andrew, Tim Barringer, Elizabeth Prettejohn (1999). Leighton and the Shifting Repertoires of “Masculine” Artistic Identity in the Late Victorian Period. Frederic Leighton: Antiquity, Renaissance, Modernity.

[R45] Stephenson Andrew (2007). Precarious Poses: The Problem of Artist Visibility and its Homosocial Performances in Late Victorian London. Visual Culture in Britain.

[R46] Sturdy Steve, Andrew Patrizo, Dawn Kemp (2006). Making Sense in the Pathology Museum. Anatomy Acts: How We Come to Know Ourselves.

[R47] Swanton Ernest W (1947). A Country Museum: The Rise and Progress of Sir Jonathan Hutchinson’s Educational Museum at Haslemere.

[R48] Thompson Henry [Pen Oliver] (1885). Charley Kingston’s Aunt.

[R49] Thompson Henry (1888). Clinical Lectures on Diseases of the Urinary Organs: Delivered at University College Hospital.

[R50] Tucker Jennifer, Lightman Bernard (1997). Photography as Witness, Detective, and Imposter: Visual Representation in Victorian Science. Victorian Science in Context.

[R51] Waddington Keir (2002). Mayhem and Medical Students: Image, Conduct, and Control in the Victorian and Edwardian London Teaching Hospital. Social History of Medicine.

[R52] Wakeley Cecil (1948). Annals of the Royal College of Surgeons of England.

[R53] Warner John Harley, Warner JH, Edmonson James M (2009). Witnessing Dissection: Photography, Medicine, and American Culture. Dissection: Photographs of a Rite of Passage in American Medicine: 1800–1930.

[R54] Yates Edmund (1878). Celebrities at Home.

